# High-Pressure Pneumoperitoneum Aggravates Surgery-Induced Neuroinflammation and Cognitive Dysfunction in Aged Mice

**DOI:** 10.1155/2020/6983193

**Published:** 2020-06-19

**Authors:** Bo Lu, Hui Yuan, Xiaojie Zhai, Xiaoyu Li, Jinling Qin, Junping Chen, Bo Meng

**Affiliations:** ^1^Department of Anesthesiology, HwaMei Hospital, University of Chinese Academy of Sciences, Ningbo 315010, China; ^2^Ningbo Institute of Life and Health Industry, University of Chinese Academy of Sciences, Ningbo 315010, China

## Abstract

Postoperative cognitive dysfunction (POCD) is a common complication after surgery, especially in aged patients. Neuroinflammation has been closely associated with the development of POCD. While the contribution of pneumoperitoneum to the systemic inflammation has been well documented, the effect of pneumoperitoneal pressure on neuroinflammation and postoperative cognitive function remains unclear. In this study, we showed that high-pressure pneumoperitoneum promoted the postoperative neuroinflammation and microglial activation in the hippocampus and aggravated the postoperative cognitive impairment in aged mice. These results support the requirement to implement interventions with lower intra-abdominal pressure, which allows for adequate exposure of the operative field rather than a routine pressure.

## 1. Introduction

Postoperative cognitive dysfunction (POCD) is characterized by deterioration in cognitive functions, mainly learning and memory, which can last from days to years. POCD can be associated with long-term disability and high healthcare costs, but also with increased mortality. Little attention has been directed to pneumoperitoneum pressure despite the growing interest in the risk factors of POCD, such as age, low educational levels, previous cerebrovascular accidents, and preoperative cognitive impairment [1, 2].

Laparoscopy has been shown to be a great surgical improvement compared with laparotomy [3]. Indeed, laparoscopy has been shown to reduce blood loss, scar formation, hospital stays, and postoperative recovery periods, compared with laparotomy [4, 5]. In this regard, a pneumoperitoneal pressure (PP) of 12-15 mmHg is currently applied in clinical settings due to the hemodynamic changes associated with higher PP levels [6]. However, the effect of PP on clinical outcomes has received less attention. Indeed, while most studies have focused on the impact of low PP (LPP) on operation conditions and postoperative pain after CO_2_ pneumoperitoneum production [7–9], few studies have assessed the impact of PP on POCD.

Schietroma et al. demonstrated that PP reduction to 6-8 mmHg during laparoscopic adrenalectomy can reduce the postoperative systemic inflammatory response [10]. In addition, studies have shown that the induction of systemic inflammatory mediators by surgical trauma is the main source of central neuroinflammation [11–15]. Of interest, neuroinflammation has been closely associated with the development of POCD [16–18]. Hence, we hypothesized that HPP promotes neuroinflammation and exacerbates the postoperative neurocognitive disorders. Therefore, the aim of this study was to determine the effects of different PPs on surgery-induced neuroinflammation and cognitive impairment.

## 2. Materials and Methods

### 2.1. Animals

Male Institute of Cancer Research (ICR) mice (12-14 months, 40-55 g), used in this study, were purchased from the Experimental Animal Center of Zhejiang Province, China. All experimental procedures involving animals were approved by the Animal Care and Use Committee of Ningbo University in accordance with the guidelines for the Care and Use of Laboratory Animals by the National Institutes of Health (NIH Publications No. 80-23). All animals were fed standard rodent food and water *ad libitum* and were housed, four mice per cage, in a temperature-controlled animal facility with 12 h light/dark cycles.

### 2.2. Anesthesia and Perioperative Management

Anesthesia was induced by 3-5% sevoflurane in a chamber with 100% oxygen. After endotracheal intubation, animals were connected to a rodent ventilator (R415, RWD Life Science, Shenzhen China) that was adjusted to a tidal volume of 200 *μ*l at 150 strokes per minute. Anesthesia was maintained using an inhalational anesthesia circuit system (R500SE, RWD Life Science, Shenzhen China).

### 2.3. Pneumoperitoneal Implementation

The mice were placed in the supine position. A 20-gauge catheter was inserted in the right-lower quadrant of the abdomen and connected to an electronic endoflator with air source (Karl Storz, Germany) at a flow rate of 0.1 l/min. The pneumoperitoneum maintenance time was 30 minutes. The low pneumoperitoneum pressure was set to 2 mmHg, while the high pneumoperitoneum pressure was set to 8 mmHg [19].

### 2.4. Surgical Procedures

Abdominal exploration was modified based on the previously described procedures [20, 21]. Briefly, a 1.5 cm incision was made below the lower-right rib, through which a 0.5 cm sterile probe was inserted into the body cavity to manipulate the viscera and musculature with a frequency of 1 per second for a period of 1 minute. After that, a 5 cm region of the intestine was exteriorized and manipulated between the surgeon's thumb and forefinger for 1 minute. The time of the operation was controlled within 15 minutes, and the mice were naturally awake after the termination of action of the anesthetic agent.

At the end of the surgery, analgesia was performed by subcutaneous injection of 0.1 ml of 1.0% ropivacaine into the incision area, and the wound was sutured and covered with polysporin to prevent potential infection.

### 2.5. Animal Grouping

A total of 64 mice were randomly divided into 4 groups: control group (con), surgery group (sur), surgery+low PP group (sur+LP), and surgery+high PP group (sur+HP). To avoid the possible confounding effects of behavioral tests on inflammatory markers, half of the animals in each group were subjected to the behavioral tests, while the other half were sacrificed 24 hours after surgery for ELISA and immunostaining.

### 2.6. Behavioral Tests

**Figure 1:**
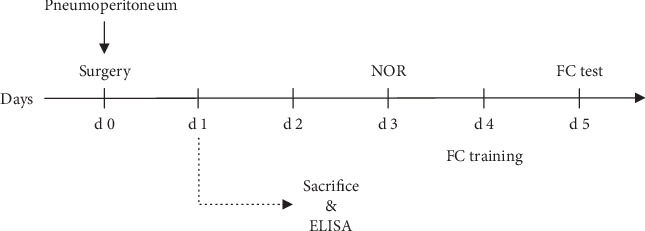
The study design. Experiment 1: animals were subjected to the novel object recognition (NOR) test on postoperative day 3 (d3), the training of fear conditioning (FC) was applied on d4, and the FC test was performed on d5. Experiment 2: animals were sacrificed 24 hours after surgery, and the hippocampus was collected for ELISA and immunostaining.

After 2 days of recovery from the surgery, behavioral tests were initiated on the third postoperative day (Figure 1). All behavioral tests were conducted in a room that was adjacent to the housing room with dim light conditions.

#### 2.6.1. Novel Object Recognition (NOR)

Novel object recognition (NOR) is based on the spontaneous tendency of rodents to spend more time exploring a novel object than a familiar one. The test was performed on postoperative day 3, and it included two sessions to assess the visual and spatial short-term memory. During the familiarization session, mice were allowed to explore for 5 minutes in an open-field box containing 2 identical objects (A and B). The mice were then allowed to rest for 4 hours. The test session was then performed for 5 min, wherein object A was replaced by a novel object C with different shape, material, and color. The path of the mice was recorded and analyzed for the amount of time taken to explore each object using an image analyzing system (Zhenghua Biologic Apparatus, Huaibei, China). The recognition index (RI) was calculated based on the following equation: RI = exploration time of the novel object/total exploration time for both objects. During the familiarization session, the average speed of movement was also recorded to assess the motor activity and exploratory activity.

#### 2.6.2. Fear Conditioning

The fear conditioning test was used to assess fear memory associated with a conditional stimulus [22]. The test was conducted using a conditioning chamber (30 × 30 × 45 cm, SuperFcs, Xinruan Information, Shanghai, China). On postoperative day 4, the mice were allowed to explore the conditioning chamber for 180 seconds before exposure to fear conditioning. The mice were then exposed to the conditional stimulus, an auditory cue for 30 s (70 dB, 3 kH), and to the unconditional stimulus, a 2-second foot shock (0.75 mA), which was administered immediately after termination of the tone. This procedure was repeated with an interval of 60 seconds. On postoperative day 5, the mice were returned into the same chamber; however, no tones or foot shocks were delivered. Mice were placed after 2 hours in a new environment (different context from training environment), and the same auditory stimulation was given for 3 minutes to test for auditory-cued memory, which reflects the hippocampal-independent fear memory. Freezing behavior, an indicator of fear memory, was measured during the exposure of mice to the conditional stimulus. The freezing time was used to assess the memory and learning abilities. A decrease of freezing time indicated impairment in these abilities.

### 2.7. Enzyme-Linked Immunosorbent Assay (ELISA)

The mice were decapitated under anesthesia, induced by sevoflurane, and the brain was quickly removed and dissected to collect the hippocampus. The samples were rinsed with cold saline solution and homogenized for the measurement of tumor necrosis factor-alpha (TNF-*α*; Cat. No.: EM001; ExCell Bio, Taicang, China), interleukin-1 beta (IL-1*β*; Cat. No.: MTA00B; R&D, Minneapolitan, USA), and interleukin-6 (IL-6; Cat. No.: EM004; ExCell Bio, Taicang, China) using ELISA. The absorbance was read at 450 nm using a microplate spectrophotometer (Thermo Inc., USA). The concentrations were calculated with reference to a standard curve that was fitted using 4 parameter logistic regression. The values were presented as picogram per milligram of tissue. The protocols were performed according to the manufacturer's instructions (R&D Systems, USA).

### 2.8. Immunohistochemistry

After anesthesia, mice were transcardially perfused with saline solution followed with 4% paraformaldehyde (PFA). The brain was then dissected out, fixed with 4% PFA overnight, and consecutively incubated for 24 hours each in 15% and 30% sucrose solutions. The brain was then frozen in an optimal cutting temperature compound (OCT; Sakura Finetek, CA, USA) and cut into 25 *μ*m thick sections (CM1950, Leica, Frankfurt, Germany). Sections containing the hippocampus were incubated overnight at 4°C in 0.1 M PBS buffer containing 0.5% TritonX-100 and goat anti-ionized calcium-binding adapter molecule 1 (Iba-1, dilution 1: 500; Abcam, Cambridge, USA). After that, sections were washed three times in PBS solution, 8-10 minutes each, then incubated for 90 minutes at room temperature in the same PBS solution containing Alexa 488-conjugated donkey anti-goat antibody (dilution 1 : 500; Abcam, Cambridge, USA). Three sections were imaged per mouse using a confocal laser scanning microscope (SP8, Leica, Frankfurt, Germany). Iba-1 staining was analyzed in a blinded manner using the ImageJ software (NIH, USA). The number of pixels per image with intensity above a predetermined threshold level was considered to be positively stained. The degree of positive immunoreactivity was reflected by the percentage of the positively stained area in the total area of the interested structure in the imaged field.

### 2.9. Statistical Analysis

Statistical analysis was performed using GraphPad Prism 8.0 (GraphPad Software, San Diego, USA). All data are expressed as mean ± standard error of the mean (SEM). Statistical comparisons were performed using one-way analysis of variance (ANOVA) followed with Bonferroni's post hoc test (con *vs.* sur, sur *vs.* sur+LP, and sur *vs.* sur+HP). *P* < 0.05 was considered statistically significant.

## 3. Results

### 3.1. HPP Enhanced the Postoperative Cognitive Impairment in Aged Mice

There was no significant difference in the average speed of movement among the four groups (*F* = 0.847, *P* > 0.05), suggesting that the motor activity and exploratory activity were not affected by the surgery.

**Figure 2:**
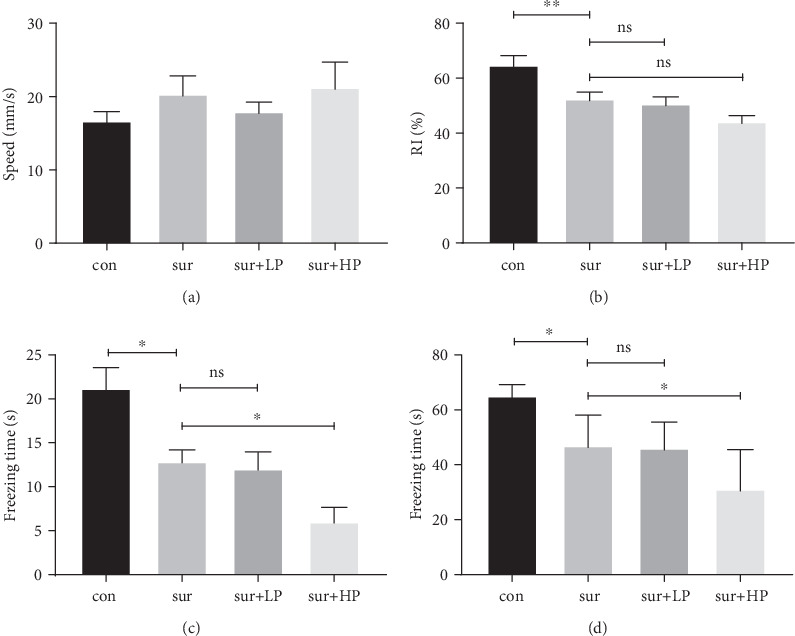
The effect of different pneumoperitoneal pressure levels on surgery-induced postoperative cognitive impairment in aged mice. (a) The average speed reflects motor activity and exploratory activity. (b) The recognition index (RI) reflects the object recognition memory. (c) Contextual fear memory reflects the hippocampal-dependent fear memory. The freezing time was used to measure the memory and learning abilities. A decrease in the freezing time indicates reduction in these abilities. (d) Cued fear memory reflects the hippocampal-independent fear memory. The data are presented as mean ± SEM (*n* = 8 per group). ∗*P* < 0.05; ∗∗*P* < 0.01.

Visual recognition memory and fear memory were assessed using NOR and FC tests, respectively, to examine the effect of different PP levels on surgery-induced cognitive impairment. While the control mice spent significantly more time exploring the novel object relative to the familiar object (*t* = 3.22, *P* < 0.01, Figure 2(b)), the surgically treated mice were unable to discriminate between the familiar and novel objects. In addition, the surgery group mice produced a hippocampus-dependent and hippocampus-independent fear memory dysfunction as evidenced by the significant decrease in their freezing time in the FC test (Contextual FC: *t* = 3.168, *P* < 0.05; Cued FC: *t* = 3.067, *P* < 0.05; Figures 2(c) and 2(d)). On the other hand, the mice showed a higher reduction in their freezing behavior when HPP was performed preoperatively, compared with the surgery group mice (Contextual FC: *t* = 2.609, *P* < 0.05; Cued FC: *t* = 2.698, *P* < 0.05). In contrast, the LPP did not affect the fear memory impairment induced by surgery (Contextual FC: *t* = 0.315, *P* > 0.05; Cued FC: *t* = 0.158, *P* > 0.05). These results suggest that HPP enhanced the surgery-induced cognitive impairment in aged mice. However, neither LPP nor HPP further aggravated the impairment of object recognition memory, compared with the surgery group (*t* = 2.167, *P* > 0.05).

### 3.2. HPP Promoted the Postoperative Neuroinflammation in the Hippocampus of Aged Mice

**Figure 3:**
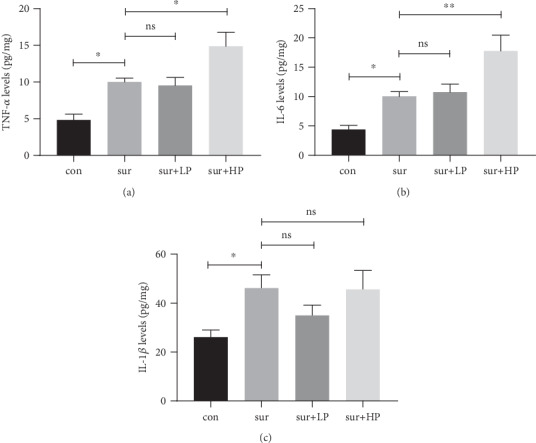
Postoperative neuroinflammation in the hippocampus. The protein expression of (a) TNF-*α*, (b) IL-6, and (c) IL-1*β* in the hippocampus. The protein levels were measured using ELISA on homogenized tissue samples. The data are presented as mean ± SEM (*n* = 4 per group). ∗*P* < 0.05; ∗∗*P* < 0.01.

Neuroinflammation is closely associated with POCD. Therefore, the levels of inflammatory cytokines in the hippocampus, including TNF-*α*, IL-6, and IL-1*β*, were measured 24 hours after surgery. Surgery induced an increase in the hippocampal expression of TNF-*α* (*t* = 3.054, *P* < 0.05, Figure 3(a)), IL-6 (*t* = 2.487, *P* < 0.05, Figure 3(b)), and IL-1*β* (*t* = 2.610, *P* < 0.05, Figure 3(c)). In addition, HPP, but not LPP, further promoted the surgery-induced increase in hippocampal expression of TNF-*α* (sur vs. sur+HP: *t* = 2.865, *P* < 0.05; sur vs. sur+LP: *t* = 0.275, *P* > 0.05) and IL-6 (sur vs. sur+HP: *t* = 3.407, *P* < 0.01; sur vs. sur+LP: *t* = 0.315, *P* > 0.05).

### 3.3. HPP Enhanced the Postoperative Microglial Activation in the Hippocampus of Aged Mice

**Figure 4:**
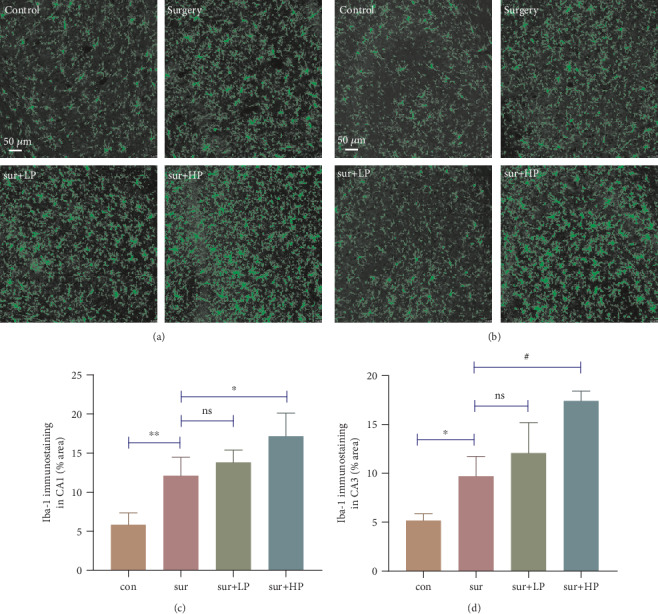
Microglial activation in the hippocampus 24 hours after surgery. (a, b) Iba-1 immunostaining in the CA1 and CA3 regions of the hippocampus. Scale bar 50 *μ*m. (c, d) Quantification of Iba-1-positive cells in the CA1 and CA3 regions of the hippocampus. Data are expressed as mean ± SEM (*n* = 4 per group). ∗*P* < 0.05; ∗∗*P* < 0.01; ^#^*P* < 0.001.

Due to the important role of microglia in the development of neuroinflammation during CNS disorders, we aimed to assess microglial activation in the CA1 and CA3 regions of the hippocampus 24 hours after surgery by detecting the marker of microglia, Iba-1, using immunostaining. As shown in Figure 4, mice in the surgery group showed higher number of Iba-1-positive cells in the CA1 and CA3 regions, compared with those in the control group (CA1: *t* = 4.039, *P* < 0.01; CA3: *t* = 3.248, *P* < 0.05). Compared with the surgery group, a higher percentage of Iba-1-positive cells was observed in the HPP group, but not in the LPP group (sur vs. sur+HP: CA1: *t* = 3.263, *P* < 0.05; CA3: *t* = 5.544, *P* < 0.001; sur vs. sur+LP: *t* = 1.103, *P* > 0.05; CA3: *t* = 1.710, *P* > 0.05).

## 4. Discussion

Laparoscopic surgery presents several advantages over laparotomy, such as reduced postoperative pain, prompt postoperative bowel activity, reduced hospitalization, rapid recovery, better aesthetic results, and reduced postoperative infections [4, 5]. Despite that laparoscopy is considered to be “minimally invasive,” the required pneumoperitoneum during laparoscopy can cause mechanical damage by expansion of the abdominal wall through positive pressure. This can lead to a significant reduction in blood perfusion of abdominal organs and induction of anaerobic metabolism, which can lead to lactic acidosis, oxidative stress, and organ damage [23, 24]. In fact, few studies have assessed the impact of pneumoperitoneum on the perioperative neurocognitive disorders. Here, we showed that the abdominal exploration surgery can impair object recognition memory and hippocampus-dependent and hippocampus-independent fear memory. In addition, we showed that HPP during the surgery can further exacerbate the surgery-induced impairment in fear memory.

Neuroinflammation has been associated with surgery-induced cognitive dysfunction. The release of proinflammatory cytokines, such as TNF-*α* and IL-1*β*, has been reported as a critical factor in the development of cognitive deficits [17, 25]. Indeed, changes in the levels of proinflammatory cytokines in the cerebrospinal fluid of postsurgical patients have been shown to play a role in the neuroinflammatory response during POCD pathophysiology [11, 26]. In addition, activated microglia were shown to induce the overproduction of proinflammatory cytokines, which contributes to long-term neuroinflammation. In this study, we showed that abdominal surgery induced neuroinflammation and microglial activation in the hippocampus and that HPP further increased the hippocampal levels of the inflammatory factors TNF-*α* and IL-6, concomitant with enhanced microglial activation. These results suggest that the pneumoperitoneum is part of the laparoscopic surgery trauma and that high intra-abdominal pressure can promote postoperative neuroinflammation in the hippocampus.

The blood-brain barrier (BBB) regulates the movement of biomolecules into and out of the brain. Disruption of the BBB function can trigger a transient or chronic leakage of plasma components into the brain tissue, which can lead to a disruption in the brain's homeostasis and triggering a pathological state [27, 28]. It has been demonstrated that anesthesia and surgery can induce an age-associated dysfunction of BBB in mice, concomitant with cognitive impairment [12, 29]. Schietroma et al. investigated the effect of high and low pneumoperitoneal pressure (12-14 mmHg vs. 6-8 mmHg) on peripheral inflammatory biomarkers during laparoscopic adrenalectomy. The authors observed an increase in the levels of systemic inflammatory biomarkers, such as IL-1, IL-6, and CRP, after surgery in the HPP group [10]. Accordingly, we proposed that trauma caused by the pneumoperitoneum can aggravate the surgery-induced systemic inflammatory response and that systemic inflammatory factors can induce neuroinflammation in the central nervous system by passing through the impaired BBB. However, further studies are required to confirm this hypothesis.

The NOR test and the FC test, which are widely used to study POCD, were applied in this study to evaluate postoperative cognitive functions. While HPP further enhanced the surgery-induced reduction in fear memory observed in the FC test, HPP did not cause further impairment in the object recognition memory of aged mice. Nevertheless, we believe that surgical trauma can impair cognitive function in multiple aspects through different brain regions. Indeed, POCD is generally diagnosed using a multidimensional and multifaceted neuropsychiatric scale [30]; hence, animal studies should also integrate multiple behavioral tests to comprehensively evaluate their cognitive functions. While we were unable to detect a significant impact on the object recognition memory in this study, NOR test alone might not be sensitive enough to detect variability in cognitive functions.

It was shown that the neuroinflammatory response reaches a peak 24 hours after surgery [31–33]. Hence, the 24-hour time point after surgery was selected for the measurement of neuroinflammatory factors, such as cytokine levels and microglial activation. While the cytokine levels were measured at time points different from those used for behavioral measurements, we cannot guarantee that performing cytokine and behavioral measurements at the same time would have yielded different results than those obtained in this study.

This study has several limitations. First, the effect of pneumoperitoneum duration on the cognitive function and neuroinflammatory response was not evaluated. Hence, further studies are required to fully investigate this issue in animal models. Second, the effect of the pneumoperitoneum on inflammation was assessed only in the hippocampus. However, the pneumoperitoneum may also induce inflammation in other regions of the brain, such as the medial prefrontal cortex, amygdala, and the cortex. Third, laboratory measurements were focused on a single time point that previous studies have confirmed in the presence of cognitive impairment and neuroinflammation. Fourth, air was used as the source of pneumoperitoneum to exclude the effect of carbon dioxide-induced hypercapnia in this study, which is inconsistent with clinical practice. Fifth, no muscle relaxant was used in this study. We tried to simulate the clinical scenario where deep muscle relaxation was applied but found it difficult to achieve. Eventually we decided to simplify the model while we can still tell various pressures generating different levels of neuroinflammation.

In conclusion, our results showed that HPP can aggravate the surgery-induced impairment in fear memory, but also can promote surgery-induced hippocampal inflammation in aged mice. These data support the use of interventions with the lowest intra-abdominal pressure, which allow adequate exposure of the operative field rather than routine pressure. LPP under deep muscle relaxation anesthesia, generally defined as intra-abdominal pressure of 6-10 mmHg, can satisfy the operational field and surgical conditions of most laparoscopic surgeries. While most studies have focused on the benefits of LPP on surgical conditions, postoperative pain, and rapid recovery, few studies have addressed its effect on postoperative cognitive dysfunction. In fact, an increasing number of elderly patients are expected to undergo laparoscopic “major surgery.” Since age is an independent risk factor for POCD, a large number of randomized controlled clinical trials are required to study the influence of pneumoperitoneum on postoperative cognitive dysfunction.

## Data Availability

The datasets used and/or analyzed during the current study are available from the corresponding author on reasonable request.

## References

[1] Eckenhoff R. G., Maze M., Xie Z. (2020). Perioperative neurocognitive disorder: state of the preclinical science. *Anesthesiology*.

[2] Hu J., Feng X., Valdearcos M. (2018). Interleukin-6 is both necessary and sufficient to produce perioperative neurocognitive disorder in mice. *British Journal of Anaesthesia*.

[3] Kennedy G. D., Heise C., Rajamanickam V., Harms B., Foley E. F. (2009). Laparoscopy decreases postoperative complication rates after abdominal colectomy: results from the national surgical quality improvement program. *Annals of Surgery*.

[4] Keus F., de Jong J. A., Gooszen H. G., van Laarhoven C. J. (2006). Laparoscopic versus open cholecystectomy for patients with symptomatic cholecystolithiasis. *Cochrane Database of Systematic Reviews*.

[5] Nieboer T. E., Johnson N., Lethaby A. (2009). Surgical approach to hysterectomy for benign gynaecological disease. *Cochrane Database of Systematic Reviews*.

[6] Neudecker J., Sauerland S., Neugebauer E. (2002). The European Association for Endoscopic Surgery clinical practice guideline on the pneumoperitoneum for laparoscopic surgery. *Surgical Endoscopy*.

[7] Barczynski M., Herman R. M. (2003). A prospective randomized trial on comparison of low-pressure (LP) and standard-pressure (SP) pneumoperitoneum for laparoscopic cholecystectomy. *Surgical Endoscopy*.

[8] Sandhu T., Yamada S., Ariyakachon V., Chakrabandhu T., Chongruksut W., Ko-iam W. (2009). Low-pressure pneumoperitoneum versus standard pneumoperitoneum in laparoscopic cholecystectomy, a prospective randomized clinical trial. *Surgical Endoscopy*.

[9] Sarli L., Costi R., Sansebastiano G., Trivelli M., Roncoroni L. (2000). Prospective randomized trial of low-pressure pneumoperitoneum for reduction of shoulder-tip pain following laparoscopy. *The British Journal of Surgery*.

[10] Schietroma M., Pessia B., Stifini D. (2016). Effects of low and standard intra-abdominal pressure on systemic inflammation and immune response in laparoscopic adrenalectomy: a prospective randomised study. *Journal of Minimal Access Surgery*.

[11] Culley D. J., Snayd M., Baxter M. G. (2014). Systemic inflammation impairs attention and cognitive flexibility but not associative learning in aged rats: possible implications for delirium. *Frontiers in Aging Neuroscience*.

[12] Subramaniyan S., Terrando N. (2019). Neuroinflammation and perioperative neurocognitive disorders. *Anesthesia and Analgesia*.

[13] Daneman R. (2012). The blood-brain barrier in health and disease. *Annals of Neurology*.

[14] D'Mello C., Le T., Swain M. G. (2009). Cerebral microglia recruit monocytes into the brain in response to tumor necrosis factoralpha signaling during peripheral organ inflammation. *The Journal of Neuroscience*.

[15] Sweeney M. D., Sagare A. P., Zlokovic B. V. (2018). Blood-brain barrier breakdown in Alzheimer disease and other neurodegenerative disorders. *Nature Reviews Neurology*.

[16] Hovens I. B., van Leeuwen B. L., Nyakas C., Heineman E., van der Zee E. A., Schoemaker R. G. (2015). Postoperative cognitive dysfunction and microglial activation in associated brain regions in old rats. *Neurobiology of Learning and Memory*.

[17] Terrando N., Eriksson L. I., Kyu Ryu J. (2011). Resolving postoperative neuroinflammation and cognitive decline. *Annals of Neurology*.

[18] Terrando N., Monaco C., Ma D., Foxwell B. M. J., Feldmann M., Maze M. (2010). Tumor necrosis factor-alpha triggers a cytokine cascade yielding postoperative cognitive decline. *Proceedings of the National Academy of Sciences of the United States of America*.

[19] Matsuzaki S., Botchorishvili R., Jardon K., Maleysson E., Canis M., Mage G. (2011). Impact of intraperitoneal pressure and duration of surgery on levels of tissue plasminogen activator and plasminogen activator inhibitor-1 mRNA in peritoneal tissues during laparoscopic surgery. *Human Reproduction*.

[20] Barrientos R. M., Hein A. M., Frank M. G., Watkins L. R., Maier S. F. (2012). Intracisternal interleukin-1 receptor antagonist prevents postoperative cognitive decline and neuroinflammatory response in aged rats. *The Journal of Neuroscience*.

[21] Jia M., Liu W. X., Sun H. L. (2015). Suberoylanilide hydroxamic acid, a histone deacetylase inhibitor, attenuates postoperative cognitive dysfunction in aging mice. *Frontiers in Molecular Neuroscience*.

[22] Misane I., Tovote P., Meyer M., Spiess J., Ogren S. O., Stiedl O. (2005). Time-dependent involvement of the dorsal hippocampus in trace fear conditioning in mice. *Hippocampus*.

[23] Montalto A. S., Bitto A., Irrera N. (2012). CO2 pneumoperitoneum impact on early liver and lung cytokine expression in a rat model of abdominal sepsis. *Surgical Endoscopy*.

[24] Vittimberga F. J., Foley D. P., Meyers W. C., Callery M. P. (1998). Laparoscopic surgery and the systemic immune response. *Annals of Surgery*.

[25] Degos V., Vacas S., Han Z. (2013). Depletion of bone marrow-derived macrophages perturbs the innate immune response to surgery and reduces postoperative memory dysfunction. *Anesthesiology*.

[26] Tang J. X., Baranov D., Hammond M., Shaw L. M., Eckenhoff M. F., Eckenhoff R. G. (2011). Human Alzheimer and inflammation biomarkers after anesthesia and surgery. *Anesthesiology*.

[27] Abbott N. J., Patabendige A. A. K., Dolman D. E. M., Yusof S. R., Begley D. J. (2010). Structure and function of the blood-brain barrier. *Neurobiology of Disease*.

[28] Zlokovic B. V. (2008). The blood-brain barrier in health and chronic neurodegenerative disorders. *Neuron*.

[29] Hu N., Guo D., Wang H. (2014). Involvement of the blood-brain barrier opening in cognitive decline in aged rats following orthopedic surgery and high concentration of sevoflurane inhalation. *Brain Research*.

[30] Moller J. T., Cluitmans P., Rasmussen L. S. (1998). Long-term postoperative cognitive dysfunction in the elderly: ISPOCD1 study. *The Lancet*.

[31] Zhang X., Dong H., Li N. (2016). Activated brain mast cells contribute to postoperative cognitive dysfunction by evoking microglia activation and neuronal apoptosis. *Journal of Neuroinflammation*.

[32] Meng B., Li X., Lu B. (2020). The investigation of hippocampus-dependent cognitive decline induced by anesthesia/surgery in mice through integrated behavioral Z-scoring. *Frontiers in Behavioral Neuroscience*.

[33] Rosczyk H. A., Sparkman N. L., Johnson R. W. (2008). Neuroinflammation and cognitive function in aged mice following minor surgery. *Experimental Gerontology*.

